# Assessment of Response to Moderate and High Dose Supplementation of Astaxanthin in Laying Hens

**DOI:** 10.3390/ani11041138

**Published:** 2021-04-16

**Authors:** Dieudonné M. Dansou, Hao Wang, Ramdhan D. Nugroho, Weizhao He, Qingyu Zhao, Junmin Zhang

**Affiliations:** 1 State Key Laboratory of Animal Nutrition, Institute of Animal Science, Chinese Academy of Agricultural Sciences, Beijing 100193, China; donnedansou@outlook.com (D.M.D.); ramdhan.dn@gmail.com (R.D.N.); heweizhao2021@163.com (W.H.); zhaoqingyu@caas.cn (Q.Z.); zhangjunmin@caas.cn (J.Z.); 2 Scientific Observing and Experiment Station of Animal Genetic Resources and Nutrition in North China of Ministry of Agriculture and Rural Affairs, Institute of Animal Science, Chinese Academy of Agricultural Sciences, Beijing 100193, China

**Keywords:** astaxanthin, laying hen, egg yolk color, antioxidant, immunity, inflammation, high dose, moderate dose

## Abstract

**Simple Summary:**

With the increasing use of carotenoids, especially astaxanthin as a feed additive in the poultry industry, the concern about the health status of the laying hen and efficacy to improve egg quality in the case of overdosing was raised. Thus, we aimed to evaluate the effects of either moderate or high dose dietary supplementation of astaxanthin on eggs and laying hens’ health status. The results revealed that, at moderate dose increment, astaxanthin is well deposited in egg yolk, efficiently improves egg yolk color, and contributes to ameliorate the general health status of laying hens. Besides, the high dose supplementation presented positive effects on the coloration and enrichment of egg yolk and the health status of laying hens with no significant difference with the moderate doses to some extents. We concluded that it would be beneficial to add astaxanthin to laying hens feed at a moderate dose rather than high dose.

**Abstract:**

In this study, we evaluated the impact of moderate and high dose dietary supplementation of astaxanthin on production performance, quality of eggs, and health status of laying hens. The experiment involved 480 laying hens, divided into four groups of eight replicates. The different groups named A1, A2, A3, and A4 were allocated the same diet supplemented with *Haematococcus pluvialis* powder to provide 0, 21.3, 42.6, and 213.4 mg of astaxanthin per kilogram of feed, respectively. One-way ANOVA and linear and quadratic regression analysis were used to assess the differences between the groups. The results showed that the production performance of laying hens and the physical quality of eggs did not significantly differ between the groups (*p* > 0.05). Astaxanthin distribution in tissues was typical per bird, whereas the egg yolk coloration and astaxanthin concentration increased with the supplementation dose (*p* < 0.001). However, there was a decrease in concentration and coloration efficacy of astaxanthin at high dose supplementation (213.4 mg/kg) compared to moderate doses (21.3 and 42.6 mg/kg). Blood biochemical tests showed some discrepancies that were not ascribed to the effect of diets, and the increase in liver weight in the A4 group compared to others was equated with an adaptation of laying hens to the high dose supplementation. Astaxanthin improved superoxide dismutase (SOD) and glutathione peroxidase (GSH-Px) activities and diminished malondialdehyde (MDA) content in both liver and serum; meanwhile, the activities of SOD and GSH-Px in serum were similar between the moderate doses and high dose supplementation. Additionally, astaxanthin alleviated interleukin 2, 4, and 6 (IL-2, IL-4, and IL-6, respectively) in serum, showing the best effect in A3 and A4 groups. Besides, immunoglobulin G and M (IgG and IgM), as well as tumor necrosis factor-alpha and beta (TNF-α and TNF-β), were not much affected. It was concluded that although astaxanthin has no obvious adverse effect on the performance and health status of laying hens, it may not be valuable for egg fortification and health status improvement of laying hens at high dose supplementation. The high dose astaxanthin supplementation up to 213.4 mg/kg in the diet might be avoided.

## 1. Introduction

The improvement of diet composition becomes a key factor to improve the health status and welfare of animals [1] as well as to enhance productivity in livestock [2,3,4] and physical performance in athletic species [5,6,7,8]. For centuries, the color of egg yolk and meat has been the most critical criterion for chicken product choices, and carotenoids are used to improve this parameter [9]. As chickens cannot produce carotenoids, they are provided in the feed from various sources such as corn, corn gluten meal, dehydrated alfalfa meal, etc. [10,11]. It has been reported that carotenoids possess antioxidant properties and are beneficial to the fowls and humans through the consumption of carotenoid-enriched eggs [12]. Among carotenoids, astaxanthin is known for its most potent antioxidant properties [13]. Antioxidant enzymes, and immune and inflammatory response levels, significantly increased in both animals and humans after astaxanthin administration [14,15].

Unlike other carotenoids, such as beta-carotene and lycopene, that have shown pro-oxidation effect at high dose supplementation, astaxanthin was found with no pro-oxidation effect in subjects exposed to it [16,17,18,19]. Synthetic astaxanthin supplementation over 200 mg/kg in feed showed no adverse effect on fishes [20], and a study about sub-chronic toxicity of astaxanthin has shown that astaxanthin supplementation up to 200 g/kg of feed was safe for rats [21]. Furthermore, a study by Spiller and Dewell [22] on humans led to conclude that 6 mg/day consumption of astaxanthin derived from *Haematococcus pluvialis* is safe for adult humans. Likewise, the European Food Safety Authority Panel on Nutrition, Novel Foods and Food Allergens (EFSA NDA Panel) [23] stated that astaxanthin from *Haematococcus pluvialis* could be added to foods up to 0.2 mg/kg body weight without risk. On the other hand, a study by Weber et al. [24] on the tolerance of poultry against an overdose of canthaxanthin (10 times higher than the recommended dose of 8 mg/kg and 25 mg/kg for laying hens and broiler, respectively) revealed no adverse effect. In poultry, studies addressing astaxanthin were mainly implemented for either egg or meat quality and health improvement [11,25,26]. In a study by Gao et al. [27], xanthophylls (40% lutein and 60% zeaxanthin) supplementation at 20 and 40 mg/kg in the diets of laying hens was found to have enhanced antioxidant capacity of the laying hens and reduced lipid peroxidation. In the same way, astaxanthin supplementation at 0, 20, 40, 80 mg/kg in the diet was reported favorable for laying hens health status by Magnuson et al. [28]. Otherwise, other studies have implied much lower doses (0.7, 0.9, 1.1, 1.3, 2, 4, 8, and 16 mg/kg) of astaxanthin supplementation in the diet of laying hens mainly for egg coloration [10,11].

Despite the increasing use of astaxanthin in the poultry industry, a recommended dose for dietary supplementation of laying hens has not been established yet. Moreover, studies conducted on laying hens did not consider the effect of high dose supplementation of astaxanthin in the diet. Based on the studies mentioned above, we hypothesized that astaxanthin might not hamper the performance or health status of the laying hen but rather contributes to improve its general health status and egg yolk color in response to the supplementation dose. Therefore, we aimed to evaluate the effects of moderate and high dose supplementation of astaxanthin derived from *Haematococcus pluvialis* in laying hens through their production performance, quality of eggs, astaxanthin deposition in eggs and tissues, blood biochemical and hematological parameters, and internal organs development, as well as antioxidant, immunity, and inflammation status.

## 2. Materials and Methods

### 2.1. Birds, Housing, and Diets

A 12-week experiment was conducted on 480 Hy-line Brown laying hens (20-week-old) that have started laying at 17 weeks-old. All the laying hens were individually weighed and separated into four groups in a completely randomized design. Each group had eight replicates with 15 birds per replicate. All birds were housed in battery cages with 3 hens per cage. All the hens were maintained in an environment-controlled room during the entire experiment, as an electronic device was used to regulate the indoor physicochemical parameters. The average room temperature and relative humidity were maintained at about 21 °C and 60% respectively while carbon dioxide level was around 600 ppm. A photoperiod of 16 h was maintained daily throughout the experiment.

Before the trial start, all laying hens were fed with the control group diet for one week. The control group diet (basal diet, A1) was supplemented with astaxanthin microcapsules powder AstALPHYTM at 0.75 g/kg (A2), 1.5 g/kg (A3), and 7.5 g/kg (A4) to provide 21.3 ppm (21.3 mg/kg), 42.6 ppm (42.6 mg/kg), and 213.4 ppm (213.4 mg/kg) of astaxanthin in feed, respectively. The body weight of the laying hens at the onset of the feeding trial was 1.66 ± 0.02, 1.68 ± 0.03, 1.60 ± 0.02, and 1.64 ± 0.04 kg in A1, A2, A3, and A4 group, respectively. Astaxanthin microcapsules powder AstALPHYTM containing 2.84% of astaxanthin, was a bioproduct from *Haematococcus pluvialis* (Yunnan Erkang Biotechnology Co., Ltd., Kunming, China). The experimental diets and water were provided to hens on an ad libitum basis. The control group diet composition and nutritional levels are presented in Table 1.

There was no established reference dose for astaxanthin supplementation in the diet of laying hens. The dose inclusion used in the present study was based on previous reported literatures [10,11,27,28]. Thus, 21.3 and 42.6 mg/kg level of astaxanthin in feed was considered as moderate doses supplementation while 213.4 mg/kg, obtained by multiplying 21.3 mg/kg by 10 with reference to Weber et al. [24], was the high dose supplementation.

### 2.2. Production Performance

Productive performance including number of laying hens, hen-day egg production, egg weight, broken eggs, abnormal eggs, were recorded daily and feed intake was recorded biweekly during the feeding trial. Then, laying rate (LR), average egg weight (EW), daily egg mass (DEM), daily feed intake (DFI), and feed conversion ratio (FCR) were determined to assess the production performance of laying hens.

### 2.3. Egg Quality

At the end of 12 weeks, egg quality was evaluated using freshly randomly collected samples of 24 eggs per treatment (3 eggs per replicate). In summary, eggshell thickness was measured using an eggshell thickness gauge (ORKA Food Technology Co., Ltd., Ramat HaSharon, Israel) and an egg force reader (ORKA Food Technology Co., Ltd., Ramat HaSharon, Israel) served to assess eggshell strength prior to breaking of the eggs. Albumen heights, Haugh Units, and Roche yolk color fan (RYCF) values were determined using SONOVA egg quality analyzer (ORKA Food Technology Co., Ltd., Ramat HaSharon, Israel). The International Commission on Illumination (CIE) values of yolk color (L*, a*, b*) were determined with a precision colorimeter analyzer, Chromameter CR-400 (Konica Minolta Inc., Chiyoda, Japan). L* score tie in lightness, positive a* corresponds to redness, and positive b* to yellowness. The three egg yolks per replicate were thoroughly mixed and stored at −20 °C for astaxanthin determination in egg yolks.

### 2.4. Blood Collection and Blood Biochemistry and Hematology Test

At the end of the experiment, a total of 8 laying hens per group (one laying hen per replicate) were randomly selected and weighted. Thereafter, blood samples were collected from the wing vein into the serum separator tube without anticoagulant agent (Shandong Oset Medical Instruments Co., Ltd., Heze, China) and into the ethylenediaminetetraacetic acid (EDTA) tube (Jiangsu Kangjian Medical Supplies Co., Ltd., Taizhou, China). The serum was prepared with H1850R centrifuge (Hunan Xiangyi Laboratory Instrument Development Co., Ltd., Changsha, China) at 1466× *g* at 4 °C for 10 min, and the whole blood collected in the EDTA tube was kept for hematological analysis. All the blood sampling was performed between 8 a.m. and 10 a.m., and the time interval between blood collection and indices measurement did not exceed 8 h.

Serum biochemistry indices such as creatinine, blood urea nitrogen (BUN), calcium (Ca), inorganic phosphate (IP), total protein (TP), total bilirubin (TB), alanine aminotransferase (ALT), aspartate aminotransferase (AST), gamma-glutamyl transferase (GGT), alkaline phosphatase (ALP), triglycerides (TG), total cholesterol (TC), high-density lipoprotein (HDL), and low-density lipoprotein (LDL) were tested using an automatic biochemical analyzer, Kehua ZY KHB-1280, (Shanghai Kehua Bioengineering Co., Ltd., Shanghai, China). Very low-density lipoprotein (VLDL) was tested using an ELISA kit (Beijing Jinhai Keyu Biotechnology Development Co., Ltd., Beijing, China). A blood routine biochemical instrument Sysmex K4500 (Sysmex Corp., Kobe, Japan) was used to test the blood hematology parameters such as white blood cell (WBC), red blood cell (RBC), hemoglobin (Hgb), hematocrit (Hct), mean corpuscular volume (MCV), and mean corpuscular hemoglobin (MCH). These parameters were considered as key indicators to electrolyte imbalance, potential toxicity, and injury in major organs such as liver and kidney.

### 2.5. Slaughtering Activity and Visceral Coefficient

The same laying hens selected for blood sample collection were slaughtered. Blood was discharged from the neck veins of the birds after anesthesia at slaughter to ensure animal welfare while reducing animal stress to the barest minimum. After evisceration, different organs such as liver, spleen, kidney, lung, and heart were harvested and weighed. The visceral coefficient was calculated as organ weight/bird weight. The said organs were stored at −80 °C for further analysis.

### 2.6. Astaxanthin Content Analysis

Astaxanthin was tested in egg yolk by reference to Bjerkeng et al. and Du et al. with modification [29,30]. Firstly, 1 g of egg yolk was measured for each replicate. Then, 5 mL of tetrahydrofuran/methyl alcohol (ratio 1:1) was added, followed by vortex for 2 min. After that, the mixture was heated at 60 °C water bath for 20 min, vortexed again, and 5 mL of ethyl acetate was added. Then, occurred a third vortex followed by centrifugation at 1466× *g* at 4 °C for 5 min. Next, 1 mL of supernatant was taken into a 1.5 mL tube, and centrifugated at 9600× *g* at 4 °C for 10 min. Finally, the substance was aspired with a syringe and filtered through a 0.45 μm membrane into high performance liquid chromatography (HPLC) vials.

Based on egg yolk analysis data, pre-tests were conducted to determine the more accurate sample weights, dilution ratios, and vortex durations for tissue samples. Briefly, to a grinded tissue sample was added a corresponding solvent volume (tetrahydrofuran/methyl alcohol, 1:1), successively followed by vortex, water bath, and ethyl acetate addition. From the mixture, the solvent was evaporated using a gentle flow of nitrogen gas. Later, the mixture was reconstituted in mobile phase (tetrahydrofuran/methyl alcohol/ethyl acetate, 1:1:2). Finally, the solution was centrifuged and filtered into HPLC vials.

Shimadzu Prominence LC-20A (Shimadzu Corp., Kyoto Japan) was used to perform the HPLC phase. The chromatographic column (C30, 250 mm × 4.6 nm, 5 µm) was set at 25 °C. The detector wavelength was 474 nm, and the injection volume was 10 µL. The column velocity was 1.0 mL/min. The mobile phase was solvent A: 92% (methanol/tert-Butyl methyl ether, 81:15) and solvent B: 8% (ultrapure water). The test duration was 12 min. Astaxanthin content in samples (egg yolk and tissues) was determined based on the curve equation of the different isomer standards, sample curve shapes, dilution rate, and sample weights.

### 2.7. Antioxidant, Immunity, and Inflammation Status Assessment

Activities of superoxide dismutase (SOD) and glutathione peroxidase (GSH-Px) and content of malondialdehyde (MDA) were analyzed in liver and serum samples using ELISA kits (Nanjing Jiancheng Technology Co., Ltd., Nanjing, China). The coefficient of variation of both intra-assay and inter-assay were 1.7% (SOD) and 1.5% (GSH-Px and MDA). Interleukins 2, 4, and 6 (IL-2, IL-4, and IL-6), as well as immunoglobulins G and M (IgG, IgM), were tested in serum samples using ELISA kits (Beijing Solarbio Science and Technology Co., Ltd., Beijing, China). The coefficient of variation of both intra-assay and inter-assay for IgG, IgM, IL-2, IL-4, and IL-6 were less than 10%. Cytokines tumor necrosis factors alpha and beta (TNF-α and TNF-β) were tested using ELISA kits (Mlbio, Shanghai, China). For both TNF-α and TNF-β, the coefficient of variation of intra-assay and inter-assay were less than 10% and 15%, respectively. All the tests were performed according to the kit manufacturer’s instruction and the samples were analyzed in duplicate for each parameter.

### 2.8. Statistical Analysis

Excel 2016 served to computerize the data. Statistical software R (version 3.6.1, The R Foundation for Statistical Computing) was used to perform the analyses. The normality of data was tested by Shapiro–Wilk’s test and homogeneity of variance by Levene’s test. Data were analyzed by the one-way ANOVA and multiple comparisons of means were made using the Tukey method. To assess the relationship between measured parameters and astaxanthin dose level implementation, linear and quadratic regressions were performed. *p* < 0.05 indicated significant differences. Results are presented as average and standard deviation (mean ± STD).

## 3. Results

### 3.1. Production Performance and Egg Physical Quality

Production performance of laying hens and egg quality traits at the end of 12-week feeding trial were summarized and presented in Table 2. Laying rate (LR), average egg weight (EW), daily egg mass (DEM), daily feed intake (DFI), and feed conversion ratio (FCR) did not significantly differ between the groups (*p* > 0.05). Likewise, there was no significant difference (*p* > 0.05) among the groups for egg physical quality parameters such as shell thickness, shell strength, albumen height, and Haugh unit (HU).

### 3.2. Egg Yolk Color and Astaxanthin Concentration in Egg Yolk and Tissues

**Figure 1:**
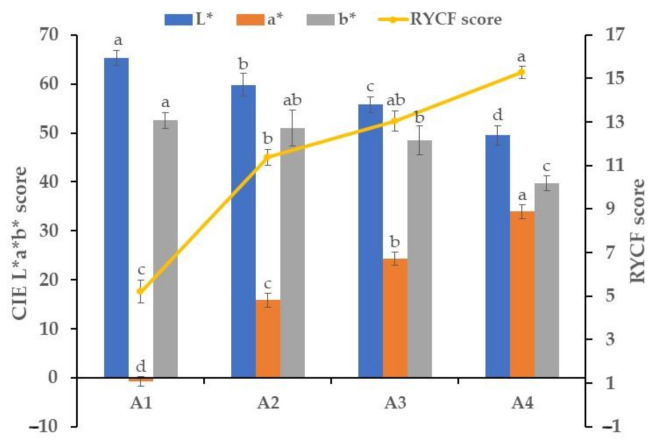
Egg yolk color score tests on week 12. Data are presented as means ± standard deviation (n = 8). a–d: different superscript letters within a same pattern indicate significant difference between groups (*p* < 0.05). A1, A2, A3, A4 = group supplemented with astaxanthin at 0 mg/kg, 21.3 mg/kg, 42.6 mg/kg, 213.4 mg/kg of diet, respectively. CIE L*a*b* score = International Commission on Illumination score where L* = lightness, a* = redness, and b* = yellowness. RYCF score = Roche yolk color fan score.

Trends of egg yolk color score are depicted in Figure 1. It shows that L* and b* scores lowered while a* score increased with astaxanthin dose augmentation in feeds. Noted that a* progression was much considerable than L* and b* regression. There was a significant difference between groups (*p* < 0.001). Concerning the RYCF values, it presented a drastic increase from 5.21 to 11.38 for A1 and A2 groups, respectively. This progress was decreased as the supplementation dose increased, trending at 13.04 for the A3 group and 15.29 for the A4 group.

Astaxanthin content in egg yolk, liver, and spleen from different layer groups was assessed and shown in Table 3. Isomers concentration and total astaxanthin in egg yolk were dependent on the supplementation dose in the diet. There were significant differences between groups (*p* < 0.001) following both linear and quadratic relationships (*p* < 0.001). Egg yolk astaxanthin contents were 9.60 µg/g in A2, 22.15 µg/g in A3, and 79.45 µg/g in A4, respectively. The highest egg yolk astaxanthin content, which was found in A4, accounted for about 8-fold the content in A2. Although overall astaxanthin content in the liver was relatively higher from one group to another, there was a considerable disproportion within the same group (standard deviation values). In contrast, the content of astaxanthin in egg yolks within a group are relatively similar. In spleen, 9-*cis* and 13-*cis* isomers in A2 and A3 were below the detection limit, but all-*trans* isomer results presented a similar trend as the one observed for liver. No astaxanthin was detected in the control group.

**Figure 2:**
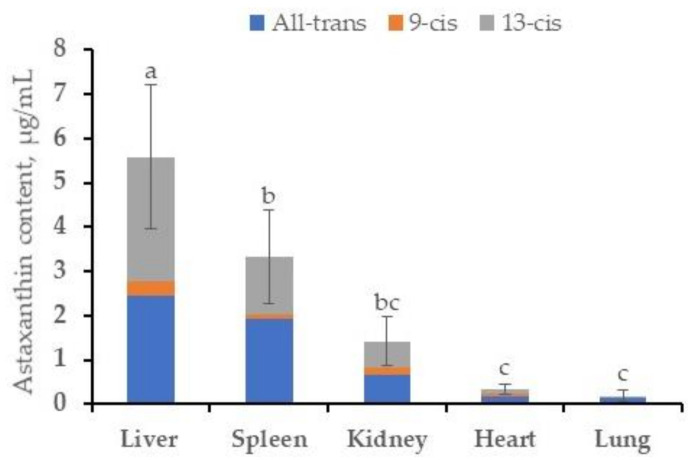
Distribution of astaxanthin in tissues of laying hens under A4 group. Data are presented as means ± standard deviation. a–c: different superscript letters indicate significant difference between groups (*p* < 0.05). A4 = laying hens supplemented 213.4 mg/kg of astaxanthin in the diet.

Apart from liver and spleen, astaxanthin distribution in kidney, lung, and heart was evaluated for laying hens under the A4 group. Results are illustrated in Figure 2. Liver exhibited the highest concentration of astaxanthin (5.27 µg/g), followed by spleen (2.97 µg/g), kidney (1.43 µg/g), heart (0.30 µg/g), and lung (0.19 µg/g).

### 3.3. Blood Test and Visceral Coefficient

To assess the susceptible changes in the internal organs of animals following diet supplementation with astaxanthin, blood biochemistry and hematology analysis was performed as shown in Table 4. Overall, the indexes did not vary much among groups. However, creatinine significantly differed between groups (*p* = 0.001). Creatinine content in A4 group blood serum (28.01 µmol/L) was higher than the content in A1, A2, and A3 (23.73, 24.33, and 23.52 µmol/L, respectively). The statistical analysis revealed linear and quadratic relationships between the groups (*p* < 0.001). Besides, ANOVA analysis exhibited a significant difference (*p* < 0.001) for ALP, that linearly (*p* = 0.027) and quadratically (*p* < 0.001) vary between the groups. A3 group presented the highest content (272.96 U/L), while the lowest content was found in A4 group (190.31 U/L). The hematological parameters did not present significant difference between the groups (*p* > 0.05).

For the visceral coefficient calculated on week 12, the body weight of laying hens and the development of their organs (spleen, heart, lung, and kidney) during the trial period were similar (Table 5). However, the laying hens under the A4 group tended to develop bigger liver than the laying hens in other groups. The statistical analyses showed significant differences (*p* < 0.001) between A4 and A1, A2, and A3, respectively.

### 3.4. Antioxidant Status

GSH-Px, SOD, and MDA were measured in liver and serum. As shown in Table 6, astaxanthin inclusion in the diet improved the antioxidant status of laying hens. This is sustained by the linear and quadratic relationship existing between the groups. In serum, GSH-Px activity was superior in all astaxanthin-supplemented diet groups compared to the control group, and regression analysis revealed a quadratic relationship between the groups (*p* = 0.014). Similarly, SOD activity was numerically superior in the astaxanthin-supplemented diet groups compared to the control group. Meanwhile, SOD activity in liver specially increased according to the supplementation dose (*p* < 0.001), and for GSH-Px activity in liver, only the A4 group showed a positive effect of astaxanthin (*p* < 0.001). Astaxanthin presented a positive impact on the MDA content as well, with a gradual decrease of MDA content in both liver and serum. The statistical analysis revealed significant differences following both linear and quadratic relationships (*p* < 0.05).

### 3.5. Immunity and Inflammation Status

Table 7 presents the immunity and inflammation status test results of laying hens on week 12. Serum IgM and IgG showed non-significant difference between the groups (*p* > 0.05). The pro-inflammatory cytokines TNF-α and TNF-β significantly differed between the groups (*p* = 0.006 and *p* < 0.001 for TNF-α and TNF-β, respectively) with the highest content in A2; meanwhile, the content in A4 was similar to that in A1. Concerning IL-2, IL-4, and IL-6, their expressions were downregulated. IL-2 and IL-6 were statistically not different between A3 and A4, but IL-4 was downregulated according to the supplementation dose.

## 4. Discussion

Previous studies on the effects of astaxanthin supplementation in diets of laying hens have shown no significant effect on their production performance [11,31]. Our results are in accordance with these previous findings. The present study reveals that even the high-level inclusion of astaxanthin (213.4 mg/kg) from *Haematoccocus pluvialis* in the diet has no adverse effect on the production performance of laying hens. Weber et al. [24] found similar results by feeding broiler, laying hens, and breeders with diets supplemented 10 times the recommended dose of canthaxanthin. Concerning the physical quality of eggs, our results indicated no particular changes between the groups. A study conducted by Cho et al. [32] involving laying hens fed diet supplemented gradual doses of canthaxanthin has revealed similar results as well. Canthaxanthin did not affect the physical quality of eggs such as Haugh Unit, eggshell strength, and eggshell thickness. Therefore, the high dose dietary supplementation of astaxanthin in laying hens does not affect the physical quality of eggs.

For decades, the use of astaxanthin from various bioproduct sources has been proven to be efficient for egg yolk coloration [33,34]. Being a red-orange carotenoid, supplementation of astaxanthin to laying hens led to a considerable increase in the redness of egg yolks and a slight decrease of lightness and yellowness in line with the findings of Johnson et al. [35] and Akiba et al. [10]. In conformity with these previous studies, we found that egg yolk color scores change with the supplementation dose level. However, there is a drastic reduction of the efficacy of astaxanthin to affect egg yolk color at high dose supplementation. The comparison of data from the moderate dose inclusions (21.3 and 42.6 mg/kg) and the high dose supplementation (213.4 mg/kg) in RYCF values and astaxanthin contents in egg yolks suggests that the egg yolk color for 213.4 mg/kg is less dependent on the content of astaxanthin in egg yolk.

The deposition of astaxanthin into egg yolk was dose level dependent. Weber et al. [24] made a similar observation with canthaxanthin accumulation in eggs of laying hens. Nevertheless, the quadratic regression result indicates that astaxanthin might be less absorbed or deposited into egg yolk at high dose supplementation. The mechanism of astaxanthin deposition into egg yolk is similar to other carotenoids, which are associated with lipid absorption and metabolism [36]. After ingestion, carotenoids are released from the food matrix, dispersed into the gastrointestinal tract thanks to dietary lipids, and then solubilized in the mixed micelles. Thereafter, the carotenoids are absorbed by the intestinal epithelial cells and are directly delivered to the liver as portomicrons. This, in contrary to mammals in which the lymphatic system is established and then involving chylomicrons [37,38]. In liver, the carotenoids are incorporated into low-density lipoproteins (LDL) and very-low-density lipoproteins (VLDL), specially very low-density lipoprotein yolk targeted (VLDLy, the specific lipoproteins for carotenoid transportation to yolk), to be transported to the ovary or other target tissues (fat, skin, etc.) by the bloodstream [12,39,40]. During this process, many factors including the consumed amount of carotenoids and dietary lipids influence the deposition of carotenoids in egg yolk. In a study on lutein supplementation to laying hens, Leeson and Caston [41] observed a decrease of lutein transfer efficiency to egg following an increased dose of lutein inclusion to diet with constant addition of oil. Later, Diwadkar-Navsariwala et al. [42] made similar observations with lycopene administration to humans. It may be concluded that the insufficient accessibility to lipids at high dose incorporation of astaxanthin limits the astaxanthin dispersion and micelles availability in the intestine, thenceforth the reduction of transfer efficiency to egg yolk.

In tissues, our results are consistent with the findings of Ytrestøyl and Bjerkeng [43]. The authors found astaxanthin more accumulated in the liver of salmon fish than other tissues. Nevertheless, the results of either Takahashi et al. [44] on broilers or Petri and Lundebye [45] on rats are not similar to ours. The inconsistency of astaxanthin distribution in tissues was earlier reported and discussed by Petri and Lundebye [45] and Waldenstedt et al. [46]. It was suspected, individual differences in the use and metabolism of carotenoids by birds. In addition, diet and astaxanthin sources, as well as sex and strain of animals, are some factors that might interfere with astaxanthin deposition in tissues. Otherwise, our observations indicate that the deposition of astaxanthin in tissues is related to each fowl, whereas the deposition into egg yolk is relatively directed by the content in feed.

Blood biochemistry test results suggest that some organs, especially the kidney and liver might misfunction in some groups [47]. The increase in serum creatinine observed in A4 is similar to the observation made by Weber et al. [24] after feeding egg breeder hens with a high dose of canthaxanthin in the diet. However, the authors have attributed the increase of creatinine in plasma to the pigmentation of plasma in high dose, which might have interfered with the photometric determination method used. Furthermore, only creatinine change does not provide a precise assessment of chicken renal function. An increase in creatinine may be the consequence of several health issues such as kidney damage, renal trauma, egg peritonitis, nephrotoxic drugs, chlamydiosis, feeding high-protein diets, etc. [48] (p. 103). Further considerations regarding the kidney would help to assess the impact of astaxanthin high dose supplementation on the said organ.

As GGT did not vary between the groups, the discrepancies observed for ALP cannot be ascribed to a dysfunction of the liver. Moreover, the highest ALP activity (272.96 U/mL) in A3 group is lower than the reference value reported by Kaneko et al. [49] (pp. 896–897). Because ALP activity in A4 was the lowest between all the groups, the increase in liver weight in A4 is not related to the variations observed for ALP as well. In fact, increases in circulating chemical enzymes may reveal hepatic hypertrophy associated with liver weight increase [50]. From a study of astaxanthin toxicity in rats, the EFSA NDA Panel [51] did not find significant differences in tissue weights of different groups. However, from carcinogenicity and chronic toxicity studies, the European Food Safety Authority Panels on Additives and Products or Substances used in Animal Feed (EFSA FEEDAP Panels) [52,53] found in female rats fed a diet supplemented astaxanthin up to 1000 mg/kg body weight, an increase in liver weight from 200 mg/kg body weight supplementation. There were appearances of hepatocellular hypertrophies and carcinoma adenomas in the livers as well. This was assigned to an adaptative metabolic process likely due to the cytochrome P450, a drug-metabolizing enzyme. Based on these reports, we might infer that the liver weight increase in our study is an adaptative response of the liver to the high dose supplementation of astaxanthin in the diet of laying hens. However, the non-correlation between the increase of serum enzyme activities and liver weight requires further studies.

Birds and other oviparous animals use carotenoids as effective immunomodulators and antioxidants which eliminate the cytotoxic reactive oxygen species (ROS) generated during normal physiological processes [54]. Analysis of astaxanthin efficacy in fishes [55,56], in rats [15], as well as in the liver, plasma, and egg yolk of laying hens [14] has shown a reduction of MDA content and increase of SOD and GSH-Px activities. Our study, as previous ones, comfort the antioxidant property of astaxanthin with the increase of GSH-Px and SOD and decrease of MDA in both liver and serum. Otherwise, this study, as many others, demonstrates the non-prooxidative effect of astaxanthin [16]. The present study shows that up to 213.4 mg/kg diet, astaxanthin remains efficient for fighting against oxidative damage in laying hens without prooxidative effect. However, though astaxanthin supplementation positively affected GSH-Px and SOD activities in serum, there was no such difference between astaxanthin-supplemented diet groups. This consideration suggests that GSH-Px and SOD activities in serum did not depend on the dose supplementation of astaxanthin. This was obvious in the results of Wang et al. [56] as well. The authors reported results of antioxidant parameters affected by the supplementation of astaxanthin and less by the dose in the diet. According to Grashorn [12], only a small proportion of carotenoids taken up by poultries is expected to be used as antioxidants, as carotenoids that are not deposited in egg yolks or tissues are broken down and excreted. The similarity of data between the moderate groups (A2 and A3) and the high group (A4) might be explained by the small amount of astaxanthin involved in free radical scavenging and singlet oxygen quenching.

Inflammation and oxidative stress processes are closely related. In fact, various inflammatory stimulants such as excessive ROS and reactive nitrogen species (RNS) produced during the oxidative metabolism as well as certain natural or artificial chemicals can trigger the inflammatory process, leading to the synthesis and secretion of proinflammatory cytokines [57]. Astaxanthin is well known to modulate the immune system and regulate the inflammatory system in both animal and human models. It has curative and preventive effects against inflammation, infectious diseases, and oxidative stress [54]. However, although some research has shown the positive effect of astaxanthin on the immune and inflammatory systems [55,58,59], other research presented no such particular changes [60,61]. In our study, regardless of the supplementation dose, astaxanthin did not affect IgM and IgG activities in serum. This is similar to the findings of Chew et al. [62] in dogs. Plasma IgM before vaccination on week 12 of the test remained unchanged and IgG was different between groups until week 6 of treatment. Despite the gradual decrease of TNF-α and TNF-β from A2 to A4, the results did not fit our expectation as the activities of the enzymes in A1 were not different from those in A4 group. Indeed, the aforementioned studies dealing with astaxanthin to improve the immune system were for the most implemented on vaccinated or challenged animals. Zhu et al. [59] claimed that fishes show defensive responses under the stimulus of external environment; then, granulocytes and white blood cells are activated to stimulate the inflammatory response and release inflammatory factors. Without challenge, immunoglobulins are less stimulated. Similarly, the immunomodulatory and anti-inflammatory properties of astaxanthin were less shown up in our study. Otherwise, the decrease of IL-2, IL-4, and IL-6 in A2, A3, and A4 groups, demonstrates the efficacy of astaxanthin to alleviate pro-inflammatory cytokines. Nevertheless, the non-significant difference of IL-2 and IL-6 between A3 and A4, indicates that the high dose supplementation might be less efficient in inflammation modulation.

## 5. Conclusions

The findings in this study revealed that the supplementation of astaxanthin in the diet does not affect production performance and egg quality of laying hens. In addition, astaxanthin has antioxidant and anti-inflammatory properties which contribute to the health status improvement of laying hens. Yet, the evaluation of egg yolk color, astaxanthin content in egg, and antioxidant property of astaxanthin in laying hens at 213.4 mg/kg supplementation demonstrated a reduction of efficacy of astaxanthin at high dose supplementation. Taken together, moderate dose supplementation of astaxanthin ensures a good egg fortification and health status of laying hens. A high dose supplementation of astaxanthin up to 213.4 mg/kg may not be recommended.

## Figures and Tables

**Table 1:** Composition and nutritional components of the control group diet.

	Items	Diet
Composition, (%)	Corn	60.92
	Soybean meal	26.65
	Soybean oil	0.60
	Limestone	9.00
	CaHPO_4_	1.00
	Premix ^1^	0.66
	DL-Met	0.17
	Zeolite powder	1.00
	Total	100.00
Nutritional components ^2^	ME, (kcal/kg)	2665
	CP, (%)	16.50
	Calcium, (%)	3.40
	Non-phytate phosphorus, (%)	0.34
	Lysine, (%)	0.86
	Methionine, (%)	0.43
	Methionine + Cystine, (%)	0.73
	Lysine/Methionine	1.98

^1^ Premix provided per kilogram of diet: vitamin A,12 500 IU; vitamin D_3_, 4125 IU; vitamin E,15 IU; vitamin K, 2 mg; vitamin B_1_, 0.98 mg; vitamin B_2_, 8.5 mg; vitamin B_6_, 8 mg; D-pantothenic acid, 50 mg; niacin, 32.5 mg; biotin, 2 mg; folic acid, 5 mg; vitamin B_12_, 5 mg; Cu, 8 mg; I, 1 mg; Fe, 60 mg; Se, 0.3 mg; Mn, 65 mg; Zn, 66 mg; phytase, 500 mg; NaCl, 3 g; choline, 500 mg. ^2^ Except ME and non-phytate phosphorus, the nutritional components are measured values.

**Table 2:** Production performance of laying hens during trial and egg physical quality of laying hens on week 12 (mean ± STD ^1^).

	Items ^4^	A1 ^2^	A2 ^2^	A3 ^2^	A4 ^2^	*p*-Value ^3^		
						AN	L	Q
Production performance	LR, (%)	86.66 ± 4.35	86.58 ± 5.20	86.67 ± 4.04	87.35 ± 5.08	0.986	0.708	0.931
	EW, (g)	57.83 ± 1.00	57.70 ± 1.48	57.48 ± 0.71	57.61 ± 0.58	0.917	0.619	0.806
	DEM, (g/hen/day)	50.15 ± 2.78	50.04 ± 2.34	49.69 ± 2.54	50.56 ± 2.94	0.931	0.791	0.781
	DFI, (g/hen/day)	115.62 ± 4.46	110.00 ± 5.25	114.84 ± 6.29	114.08 ± 5.45	0.185	0.841	0.887
	FCR, (kg/kg)	2.35 ± 0.15	2.24 ± 0.13	2.35 ± 0.20	2.30 ± 0.16	0.519	0.916	0.990
Egg physical quality	Shell thickness, (mm)	0.39 ± 0.02	0.38 ± 0.01	0.38 ± 0.02	0.40 ± 0.03	0.076	0.035	0.046
	Shell strength, (N/cm^2^)	47.54 ± 4.26	44.95 ± 3.49	42.05 ± 4.98	43.80 ± 5.35	0.127	0.371	0.567
	Albumen height, (mm)	8.34 ± 0.70	7.81 ± 1.22	7.92 ± 0.88	8.18 ± 0.66	0.62	0.797	0.548
	HU	90.41 ± 4.31	86.77 ± 7.21	87.59 ± 7.33	89.01 ± 4.97	0.65	0.894	0.593

^1^ STD = standard deviation. ^2^ A1 = basal diet; A2 = basal diet supplemented with astaxanthin at 21.3 mg/kg; A3 = basal diet supplemented with astaxanthin at 42.6 mg/kg; A4 = basal diet supplemented with astaxanthin at 213.4 mg/kg. ^3^ *p*-Value: AN = ANOVA; L = linear regression; Q = quadratic regression; differences are significant at *p* < 0.05. ^4^ Items: LR = laying rate; EW = average egg weight; DEM = daily egg mass; DFI = daily feed intake; FCR = feed conversion ratio; HU = Haugh unit.

**Table 3:** Astaxanthin content in egg yolk, liver, and spleen of laying hens on week 12 (mean ± STD ^1^).

Samples	Items ^4^	A1 ^2^	A2 ^2^	A3 ^2^	A4 ^2^	*p*-Value ^3^		
						AN	L	Q
Egg yolk, (µg/g)	All-*trans*	ND	4.55 ± 0.92 ^c^	10.26 ± 1.80 ^b^	36.70 ± 4.25 ^a^	<0.001	<0.001	<0.001
	9-*cis*	ND	0.92 ± 0.23 ^c^	1.90 ± 0.38 ^b^	5.84 ± 0.56 ^a^	<0.001	<0.001	<0.001
	13-*cis*	ND	4.14 ± 0.89 ^c^	9.99 ± 1.90 ^b^	36.91 ± 4.37 ^a^	<0.001	<0.001	<0.001
	Total	ND	9.60 ± 1.98 ^c^	22.15 ± 4.05 ^b^	79.45 ± 8.44 ^a^	<0.001	<0.001	<0.001
Liver, (µg/g)	All-*trans*	ND	0.22 ± 0.12 ^b^	0.39 ± 0.18 ^b^	2.46 ± 0.67 ^a^	<0.001	<0.001	<0.001
	9-*cis*	ND	0.03 ± 0.02 ^b^	0.05 ± 0.02 ^b^	0.31 ± 0.08 ^a^	<0.001	<0.001	<0.001
	13-*cis*	ND	0.20 ± 0.10 ^b^	0.38 ± 0.18 ^b^	2.81 ± 0.89 ^a^	<0.001	<0.001	<0.001
	Total	ND	0.44 ± 0.24 ^b^	0.82 ± 0.38 ^b^	5.58 ± 1.63 ^a^	<0.001	<0.001	<0.001
Spleen, (µg/g)	All-*trans*	ND	0.10 ± 0.05 ^b^	0.28 ± 0.20 ^b^	1.92 ± 0.61 ^a^	<0.001	<0.001	<0.001
	9-*cis*	ND	BL	BL	0.11 ± 0.05			
	13-*cis*	ND	BL	BL	1.29 ± 0.42			
	Total	ND	BL	BL	3.32 ± 1.06			

^a–c^ Different superscripts within a row indicate a significant difference (*p* < 0.05). ^1^ STD = standard deviation. ^2^ A1 = basal diet; A2 = basal diet supplemented with astaxanthin at 21.3 mg/kg; A3 = basal diet supplemented with astaxanthin at 42.6 mg/kg; A4 = basal diet supplemented with astaxanthin at 213.4 mg/kg; ND = not detected; BL = below detectable limit. ^3^ *p*-Value: AN = ANOVA; L = linear regression; Q = quadratic regression. ^4^ Items: all-*trans* = astaxanthin all-*trans* isomer content; 9-*cis* = astaxanthin 9-*cis* isomer content; 13-*cis* = astaxanthin 13-*cis* isomer content; total = all-*trans* + 9-*cis* + 13-*cis*.

**Table 4:** Serum biochemistry and blood hematology test of laying hens on week 12 (mean ± STD ^1^).

Tests	Items ^4^	A1 ^2^	A2 ^2^	A3 ^2^	A4 ^2^	*p*-Value ^3^		
						AN	L	Q
Serum biochemistry	Creatinine, (µmol/L)	23.73 ± 1.97 ^b^	24.33 ± 1.69 ^b^	23.52 ± 2.58 ^b^	28.01 ± 2.25 ^a^	0.001	<0.001	<0.001
	BUN, (µmol/L)	0.64 ± 0.05	0.65 ± 0.07	0.61 ± 0.08	0.66 ± 0.09	0.557	0.593	0.577
	Ca, (µmol/L)	3.04 ± 0.17	2.89 ± 0.06	3.03 ± 0.18	3.05 ± 0.21	0.220	0.403	0.649
	IP, (µmol/L)	1.56 ± 0.26	1.68 ± 0.19	1.43 ± 0.21	1.67 ± 0.21	0.164	0.374	0.4
	TP, (g/L)	30.43 ± 1.05	29.49 ± 0.46	31.07 ± 2.19	31.10 ± 1.82	0.147	0.198	0.399
	TB, (µmol/L)	17.97 ± 0.64	17.88 ± 0.37	17.90 ± 0.73	18.11 ± 0.55	0.864	0.460	0.701
	ALT, (U/L)	17.20 ± 1.96	17.75 ± 3.44	16.91 ± 3.62	17.33 ± 2.09	0.95	0.999	0.986
	AST, (U/L)	57.41 ± 4.67	55.40 ± 2.58	56.84 ± 4.40	55.84 ± 3.67	0.728	0.624	0.855
	GGT, (U/L)	14.39 ± 2.62	15.43 ± 2.14	15.94 ± 2.29	16.65 ± 2.77	0.334	0.118	0.18
	ALP, (U/L)	224.13 ± 13.23 ^b^	193.31 ± 11.56 ^c^	272.96 ± 23.85 ^a^	190.31 ± 15.77 ^c^	<0.001	0.027	<0.001
	TG, (mmol/L)	1.19 ± 0.27	1.37 ± 0.20	1.16 ± 0.22	1.28 ± 0.13	0.228	0.702	0.911
	TC, (mmol/L)	3.56 ± 0.44	3.74 ± 0.33	3.62 ± 0.27	3.86 ± 0.57	0.499	0.186	0.42
	HDL, (mmol/L)	0.95 ± 0.15	1.02 ± 0.11	0.98 ± 0.09	1.06 ± 0.20	0.486	0.183	0.416
	LDL, (mmol/L)	1.65 ± 0.31	1.63 ± 0.09	1.72 ± 0.26	1.78 ± 0.32	0.658	0.247	0.502
	VLDL, (mmol/L)	1.70 ± 0.29	1.56 ± 0.23	1.71 ± 0.33	1.57 ± 0.33	0.611	0.493	0.787
Blood hematology	WBC, (×10^9^/L)	73.18 ± 2.74	73.12 ± 3.06	74.92 ± 1.77	75.00 ± 1.38	0.352	0.204	0.266
	RBC, (×10^12^/L)	2.97 ± 0.29	2.74 ± 0.11	2.77 ± 0.21	2.92 ± 0.11	0.2	0.487	0.164
	Hgb, (g/L)	66.20 ± 4.66	64.80 ± 3.03	64.00 ± 2.00	67.33 ± 2.25	0.286	0.177	0.144
	Hct, (%)	30.34 ± 2.36	29.14 ± 1.48	29.45 ± 1.01	30.17 ± 1.35	0.572	0.592	0.506
	MCV, (fL)	122.98 ± 1.74	121.74 ± 3.28	121.55 ± 2.58	121.63 ± 2.49	0.785	0.616	0.611
	MCH, (pg)	24.90 ± 1.06	24.60 ± 0.76	24.20 ± 0.53	25.20 ± 0.78	0.286	0.172	0.092

^a–c^ Different superscripts within a row indicate a significant difference (*p* < 0.05). ^1^ STD = standard deviation. ^2^ A1 = basal diet; A2 = basal diet supplemented with astaxanthin at 21.3 mg/kg; A3 = basal diet supplemented with astaxanthin at 42.6 mg/kg; A4 = basal diet supplemented with astaxanthin at 213.4 mg/kg. ^3^ *p*-Value: AN = ANOVA; L = linear regression; Q = quadratic regression. ^4^ Items: BUN = blood urea nitrogen; Ca = calcium; IP = inorganic phosphate; TP = total protein; TB = total bilirubin; ALT = alanine aminotransferase; AST = aspartate aminotransferase; GGT = gamma-glutamyl transferase; ALP = alkaline phosphatase; TG = triglycerides; TC = total cholesterol; HDL = high density lipoprotein; LDL = low density lipoprotein; VLDL = very low-density lipoprotein; WBC = white blood cell; RBC = red blood cell = Hgb = hemoglobin; Hct = hematocrit; MCV = mean corpuscular volume; MCH = mean corpuscular hemoglobin.

**Table 5:** Visceral coefficient of laying hens on week 12 (mean ± STD ^1^).

Items	A1 ^2^	A2 ^2^	A3 ^2^	A4 ^2^	*p*-Value ^3^		
					AN	L	Q
Layer, (g)	1971.50 ± 88.69	1988.25 ± 126.76	1906.50 ± 238.35	1940.25 ± 122.55	0.947	0.698	0.709
Liver, (%)	1.95 ± 0.14 ^b^	2.03 ± 0.27 ^b^	1.89 ± 0.14 ^b^	2.40 ± 0.36 ^a^	<0.001	<0.001	<0.001
Spleen, (%)	0.11 ± 0.03	0.10 ± 0.02	0.10 ± 0.02	0.11 ± 0.03	0.302	0.218	0.163
Heart, (%)	0.33 ± 0.03	0.32 ± 0.02	0.33 ± 0.04	0.32 ± 0.03	0.719	0.605	0.811
Lung, (%)	0.17 ± 0.04	0.16 ± 0.02	0.16 ± 0.04	0.18 ± 0.07	0.770	0.485	0.702

^a,b^ Different superscripts within a row indicate a significant difference (*p* < 0.05). ^1^ STD = standard deviation. ^2^ A1 = basal diet; A2 = basal diet supplemented with astaxanthin at 21.3 mg/kg; A3 = basal diet supplemented with astaxanthin at 42.6 mg/kg; A4 = basal diet supplemented with astaxanthin at 213.4 mg/kg. ^3^ *p*-Value: AN = ANOVA; L = linear regression; Q = quadratic regression.

**Table 6:** Oxidation parameters of laying hens tested in liver and serum on week 12 (mean ± STD ^1^).

Items ^4^	Samples	A1 ^2^	A2 ^2^	A3 ^2^	A4 ^2^	*p*-Value ^3^		
						AN	L	Q
GSH-Px	Liver, (U/mL)	26.38 ± 3.53 ^b^	26.23 ± 3.44 ^b^	27.19 ± 2.30 ^b^	34.46 ± 4.34 ^a^	<0.001	<0.001	<0.001
	Serum, (U/mgprot)	1568.15 ± 329.39 ^b^	2167.87 ± 129.85 ^a^	2040.28 ± 244.68 ^a^	2161.31 ± 432.19 ^a^	0.004	0.069	0.014
SOD	Liver, (U/mgprot)	1298.14 ± 104.16 ^b^	1348.51 ± 79.13 ^b^	1438.12 ± 108.59 ^ab^	1595.31 ± 156.93 ^a^	<0.001	<0.001	<0.001
	Serum, (U/mL)	252.40 ± 37.30	288.33 ± 27.05	281.88 ± 62.41	286.97 ± 51.51	0.376	0.386	0.336
MDA	Liver, (nmol/mgprot)	0.58 ± 0.08 ^a^	0.50 ± 0.08 ^ab^	0.46 ± 0.05 ^ab^	0.40 ± 0.04 ^b^	0.017	0.008	0.006
	Serum, (nmol/mL)	3.86 ± 0.87 ^a^	3.62 ± 0.49 ^a^	3.12 ± 0.68 ^ab^	2.69 ± 0.36 ^b^	0.008	0.002	0.003

^a,b^ Different superscripts within a row indicate a significant difference (*p* < 0.05). ^1^ STD = standard deviation. ^2^ A1 = basal diet; A2 = basal diet supplemented with astaxanthin at 21.3 mg/kg; A3 = basal diet supplemented with astaxanthin at 42.6 mg/kg; A4 = basal diet supplemented with astaxanthin at 213.4 mg/kg. ^3^ *p*-Value: AN = ANOVA; L = linear regression; Q = quadratic regression. ^4^ Items: GSH-Px = glutathione peroxidase; SOD = superoxide dismutase; MDA = malondialdehyde.

**Table 7:** Immunity and inflammation indexes in the serum of laying hens on week 12 (mean ± STD ^1^).

Items ^4^	A1 ^2^	A2 ^2^	A3 ^2^	A4 ^2^	*p*-Value ^3^		
					AN	L	Q
IgM, (ng/mL)	29.62 ± 2.38	30.25 ± 3.09	30.61 ± 1.58	27.88 ± 2.82	0.166	0.051	0.075
IgG, (ng/mL)	920.57 ± 76.41	937.96 ± 37.96	968.68 ± 82.86	956.44 ± 52.70	0.495	0.42	0.312
TNF-α, (pg/mL)	21.45 ± 2.05 ^b^	27.64 ± 4.38 ^a^	23.71 ± 3.34 ^ab^	21.98 ± 3.34 ^b^	0.006	0.336	0.179
TNF-β, (pg/mL)	14.25 ± 1.15 ^b^	20.19 ± 2.50 ^a^	19.31 ± 1.73 ^a^	15.43 ± 2.36 ^b^	<0.001	0.410	<0.001
IL-2, (ng/mL)	2.95 ± 0.43 ^a^	2.71 ± 0.30 ^ab^	2.48 ± 0.25 ^b^	2.54 ± 0.37 ^ab^	0.049	0.145	0.018
IL-4, (pg/mL)	142.13 ± 19.81 ^a^	136.50 ± 25.27 ^ab^	132.75 ± 17.16 ^ab^	113.04 ± 12.67 ^b^	0.044	0.004	0.016
IL-6, (pg/mL)	150.85 ± 15.70 ^a^	156.68 ± 37.93 ^a^	106.59 ± 24.81 ^b^	103.97 ± 21.24 ^b^	<0.001	0.009	0.014

^a,b^ Different superscripts within a row indicate a significant difference (*p* < 0.05). ^1^ STD = standard deviation. ^2^ A1 = basal diet; A2 = basal diet supplemented with astaxanthin at 21.3 mg/kg; A3 = basal diet supplemented with astaxanthin at 42.6 mg/kg; A4 = basal diet supplemented with astaxanthin at 213.4 mg/kg. ^3^ *p*-Value: AN = ANOVA; L = linear regression; Q = quadratic regression. ^4^ Items: IgM = immunoglobin M; IgG = immunoglobin G; TNF-α = tumor necrosis factor-α; TNF-β = tumor necrosis factor-α; IL-2 = interleukin 2; IL-4 = interleukin 4; IL-6 = interleukin 6.

## Data Availability

The data presented in this study are not publicly available due to privacy restrictions.
